# Rapid Generation of Marker-Free *P*. *falciparum* Fluorescent Reporter Lines Using Modified CRISPR/Cas9 Constructs and Selection Protocol

**DOI:** 10.1371/journal.pone.0168362

**Published:** 2016-12-20

**Authors:** Catherin Marin Mogollon, Fiona J. A. van Pul, Takashi Imai, Jai Ramesar, Séverine Chevalley-Maurel, Guido M. de Roo, Sabrina A. J. Veld, Hans Kroeze, Blandine M. D. Franke-Fayard, Chris J. Janse, Shahid M. Khan

**Affiliations:** 1 Leiden Malaria Research Group, Department of Parasitology, Leiden University Medical Center (LUMC), Leiden, The Netherlands; 2 Department of Hematology, Leiden University Medical Center (LUMC), Leiden, The Netherlands; Universitatsklinikum Aachen, GERMANY

## Abstract

The CRISPR/Cas9 system is a powerful genome editing technique employed in a wide variety of organisms including recently the human malaria parasite, *P*. *falciparum*. Here we report on further improvements to the CRISPR/Cas9 transfection constructs and selection protocol to more rapidly modify the *P*. *falciparum* genome and to introduce transgenes into the parasite genome without the inclusion of drug-selectable marker genes. This method was used to stably integrate the gene encoding GFP into the *P*. *falciparum* genome under the control of promoters of three different *Plasmodium* genes (*calmodulin*, *gapdh* and *hsp70*). These genes were selected as they are highly transcribed in blood stages. We show that the three reporter parasite lines generated in this study (GFP@*cam*, GFP@*gapdh* and GFP@*hsp70*) have *in vitro* blood stage growth kinetics and drug-sensitivity profiles comparable to the parental *P*. *falciparum* (NF54) wild-type line. Both asexual and sexual blood stages of the three reporter lines expressed GFP-fluorescence with GFP@*hsp70* having the highest fluorescent intensity in schizont stages as shown by flow cytometry analysis of GFP-fluorescence intensity. The improved CRISPR/Cas9 constructs/protocol will aid in the rapid generation of transgenic and modified *P*. *falciparum* parasites, including those expressing different reporters proteins under different (stage specific) promoters.

## Introduction

A wide variety of transgenic parasite lines have been generated in rodent malaria parasites, including those that express fluorescent and/or luminescent reporter proteins under the control of constitutive or stage-specific promoters. Such transgenic ‘reporter’ parasites have proven to be useful tools to interrogate *Plasmodium* gene function, examine the effect of inhibitors on parasite development, to evaluate sub-unit vaccine efficacy *in vivo* and to rank order and evaluate live-attenuated parasite vaccines [1–12]. For rodent malaria parasites technologies have been developed to stably introduce transgenes into the parasite genome and efficient and rapid methods exist for the generation of reporter parasite lines that do not contain drug-selectable markers [13, 14]. Such ‘marker-free’ parasites make it considerably easier to further genetically modify transgenic parasites and, moreover, they can be used for drug-sensitivity testing, as possible interference from an introduced drug-selection marker is absent. In rodent malaria parasites such reporter parasite lines have been generated in multiple strains of three different *Plasmodium* species [15].

In comparison to rodent malaria parasites the technologies to genetically modify the human malaria parasite, *P*. *falciparum*, are much less efficient [16] and the number of stable reporter parasite lines in different *P*. *falciparum* strains is limited [17, 18]. In addition, currently no cloned reporter lines have been published that are drug-selectable marker free. The traditional approaches to engineer the *P*. *falciparum* genome have been hampered by the limited methods available and transfection inefficiencies in introducing exogenous DNA into the parasite genome. Also the limited number of drug-selectable markers restricts genetic engineering of *P*. *falciparum*, for example, performing sequential genetic manipulations in the same parasite line. Several technologies have been developed for the removal (re-cycling) of drug-selectable markers from the modified parasite genome, specifically using either FLP or Cre recombinases [19, 20]. However, the application of these techniques are time consuming as it can take 4–5 months to generate cloned ‘marker-free’ genetically modified parasites.

The RNA-guided CRISPR/Cas9 (clustered regularly interspaced short palindromic repeats/CRISPR-associated protein 9) system has transformed genome editing in a wide variety of organisms [21]. This powerful genome editing technique has also been applied to *P*. *falciparum* and provides an efficient method to manipulate the parasite’s genome, such as site directed mutagenesis, gene disruption and the introduction of transgenes [22, 23]. The CRISPR/Cas9 method is based on the initial generation of site-specific double strand DNA break induced by a Cas9 endonuclease and subsequent repair and modification of the DNA locus. The Cas9 enzyme is guided to a specific site in the genome by a single guide RNA (sgRNA) that can be modified to specify the exact DNA sequence within the genome. The presence of a template or ‘donor DNA’ that contains sequences surrounding the double stranded DNA break can result in guided (or homology directed) repair, resulting of introduction of donor DNA at the site of the break [24]. Frequently a two plasmid approach is used to introduce Cas9, the single guide RNA (sgRNA) composed of a fusion between CRISPR RNA (crRNA) and trans-activating CRISPR RNA (tracrRNA), and donor DNA into the nucleus of the organism. *P*. *falciparum* transfections have been performed with Cas9 and sgRNA either expressed on two separate plasmids or combined on one plasmid and different selectable markers have been used to maintain the plasmids in transformed parasites after transfection [22, 23, 25–27]. The selectable markers used are human dihydrofolate reductase (h*dhfr*), blasticidin S deaminase (*bsd*), neomycin phosphotransferase (*neo*) and yeast cytosine deaminase/uridyl phosphoribosyl transferase (y*fcu*). Generation of *P*. *falciparum* transgenic reporter parasites would benefit from the availability of standard CRISPR/Cas9 plasmids that permit the rapid introduction of different transgenes into the parasite genome without permanently integrating a drug-selectable marker cassette. Recently improved CRISPR/Cas9 constructs have been reported for marker-free editing of the *P*. *falciparum* genome [26]. One plasmid contains Cas9, the sgRNA and a *bsd* selectable marker cassette, whereas the other construct, containing the donor DNA, does not encode a drug selectable marker. The use of this ‘marker-free’ construct thus can permit an introduction of larger donor DNA sequences. Using these constructs marker-free GFP-expressing parasites have been reported.

In this paper we describe the generation of marker-free reporter parasites by using modified CRISPR/Cas9 constructs compared to the constructs described in previous studies. We generated a standard plasmid that encodes Cas9 and contains the *bsd* selection marker cassette. The sgRNA and donor DNA are both present on a second plasmid, which contains the positive selectable marker, h*dhfr*, fused to the negative selectable marker, y*fcu*. This dual positive-negative selectable marker cassette is not integrated into the genome but is used to rapidly select ‘marker-free’ transgenic parasites by the successive application of positive drug selection followed by negative selection. By generating three reporter lines which stably express GFP under the control of different promoters we show that cloned marker-free reporter parasite lines can be obtained within a period of 10–12 weeks. In addition, we show that these reporter parasites have the same *in vitro* blood-stage growth kinetics and drug-sensitivity profiles as the parental wild-type parasites and we compared the relative strengths of the different promoters to drive GFP expression. The constructs and selection protocol described in this study provide a simple set of tools to rapidly generate modified *P*. *falciparum* lines, in particular transgenic parasites that can be used to examine different *Plasmodium* regulatory elements to control transgene expression. The same constructs can be used to perform other genetic modifications, for example gene disruption or gene mutation, to interrogate gene function and can be used to perform rapid and multiple successive genetic manipulations.

## Results

### Improved CRISPR/Cas9 plasmids to introduce transgenes into the Plasmodium genome without the addition of drug selectable markers

**Fig 1:**
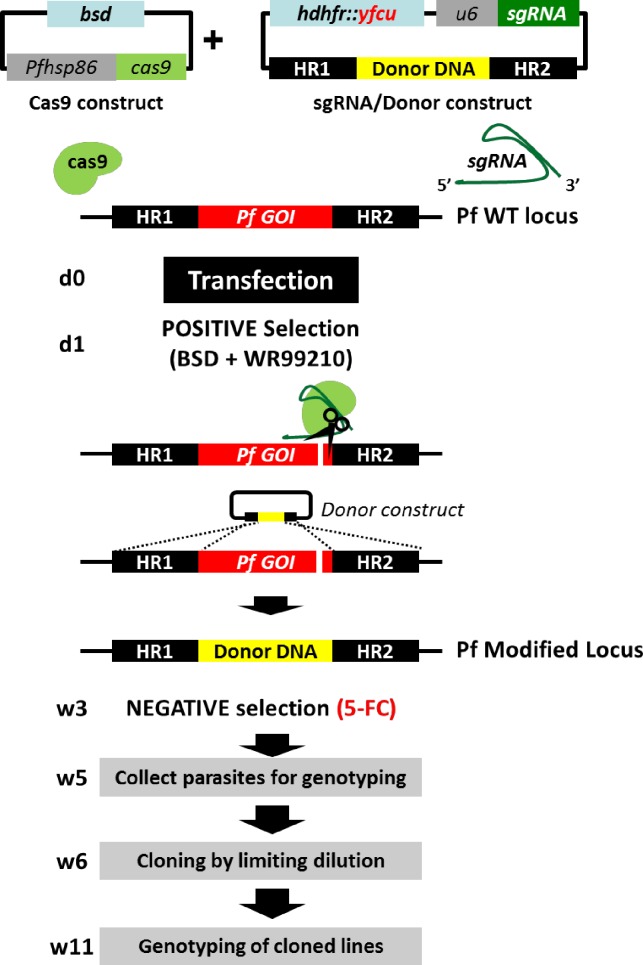
Schematic representation of improved CRISPR/Cas9 plasmids and selection protocol. Parasites are transfected with two plasmids (Cas9 construct and sgRNA/donor construct). The Cas9 construct contains the *bsd* selectable marker. The sgRNA/donor construct contains a fusion of the positive selectable marker h*dhfr* and the negative selectable marker y*fcu* genes and two homology regions (HR1 and HR2) that target a gene of interest (GOI) and introduce the donor DNA by homologous recombination. Double positive selection using both BSD and WR99210 is applied from day (d) 1 resulting in the selection of parasites that contain both plasmids within a period of 3 weeks (w). After positive selection, cultures are maintained 2–4 days without drug before negative selection is applied using 5-FC to select parasites free of episomal plasmid DNA. Parasites are genotyped by diagnostic PCR for integration of the donor DNA followed by cloning of the parasites by limiting dilution (w6). Clones are genotyped for the correct genotype by diagnostic PCR and Southern analysis. This transfection and selection protocol can result in the generation of cloned mutant parasites within a period of 11 weeks.

Homologous recombination using the CRISPR/Cas9 protocol requires introduction of the Cas9 endonuclease complexes with a sgRNA and DNA sequences (donor DNA) that will induce a double stranded break in the genome and then repair the target region. Often these different elements are present on two different plasmids encoding different drug-selectable markers. To introduce Cas9 we used the plasmid described by Ghorbal *et al*. [22]. However, in this construct (pLf0019) we replaced the y*dhodh* drug-selectable marker (SM) by the more standardly used *bsd* SM (Fig 1) as the drug Blasticidin (BSD) is easier to obtain than DSM1 that is used in conjunction with the y*dhodh* SM.

A second plasmid (pLf0022) was generated that both contains the donor DNA, the sgRNA expression cassette and drug-selectable marker. For sgRNA we used the expression cassette of the published plasmid pL6-eGFP [22], which contains the BtgZ1 adaptor sequence and the tracrRNA sequence under control of the *Plasmodium u6* RNA promoter. The drug-selectable marker cassette we used is a fusion of the positive selectable marker h*dhfr* (human dihydrofolate reductase), and the negative selectable marker y*fcu* (*yeast cytosine deaminase/uridyl phosphoribosyl transferase*) [28]. This fusion gene, h*dhfr*::y*fcu*, was placed under control of the *P*. *falciparum hsp86* promoter and the *P*. *berghei dhfr/ts* transcriptional terminator (3’UTR) and the positive-negative drug selection marker was tested in a transient transfection drug-sensitivity assay. In this assay, parasites of the *P*. *falciparum* NF54 line were transiently transfected with a circular plasmid (pLf0033; S1A Fig) encoding the h*dhfr*::y*fc* fusion protein and were treated with either WR99210 (positive) or 5-FC (negative) for 12–16 days. The transiently transfected parasites treated with WR99210 exhibit a growth rate comparable to untreated NF54 wild type parasites S1B Fig), whereas treatment with negative drug selection (using 5-FC) killed transfected parasites (S1C Fig). The h*dhfr*::y*fcu* fusion cassette therefore confers both resistance to WR99210 and sensitivity to 5-FC and can be used efficiently for positive and negative selection in *P*. *falciparum* transfections. We reasoned that by applying first positive selection (with BSD and WR99210) followed by negative selection (with 5-Fluorocytosine, 5-FC), would improve the selection of parasites where the donor DNA had been integrated into the genome. Specifically, applying first positive selection will select for parasites that contained both plasmids, resulting in a DNA break followed by donor DNA mediated repair of the target locus. Once parasites were visible in blood stage cultures we applied negative selection to select only parasites free of episomal plasmid DNA. Moreover, we placed the selectable marker fusion cassette outside the donor DNA cassette in the plasmid (Fig 1). This location permits the introduction of donor DNA sequences into the target locus without the introduction of a drug-selectable marker into the parasite genome (Fig 1). The fusion of the positive and negative selectable marker cassette also reduces the size of the overall plasmid compared to a construct where both selectable markers were controlled by separate regulatory elements. This increases the size of heterologous DNA that can be introduced as donor DNA; in S2 Fig the plasmid maps of both the Cas9 (pLf0019) and the sgRNA/Donor (pLf0022) are shown in more detail.

### Generation of (donor DNA) constructs to introduce different GFP-expression cassettes into the *P*. *falciparum* genome

To introduce transgenes into the *P*. *falciparum* genome we further modified pLf0022, and introduced homology sequences to target the *P*. *falciparum p230p* (*Pf230p*) gene locus. The *Pf230p* gene is not transcribed in asexual blood stages [29] and is therefore unlikely to be essential for asexual blood stage development/multiplication. Homology region 1 (HR1) and homology region 2 (HR2) were both PCR amplified from *P*. *falciparum* (NF54) genomic DNA and cloned into plasmid pLf0022.

**Fig 2:**
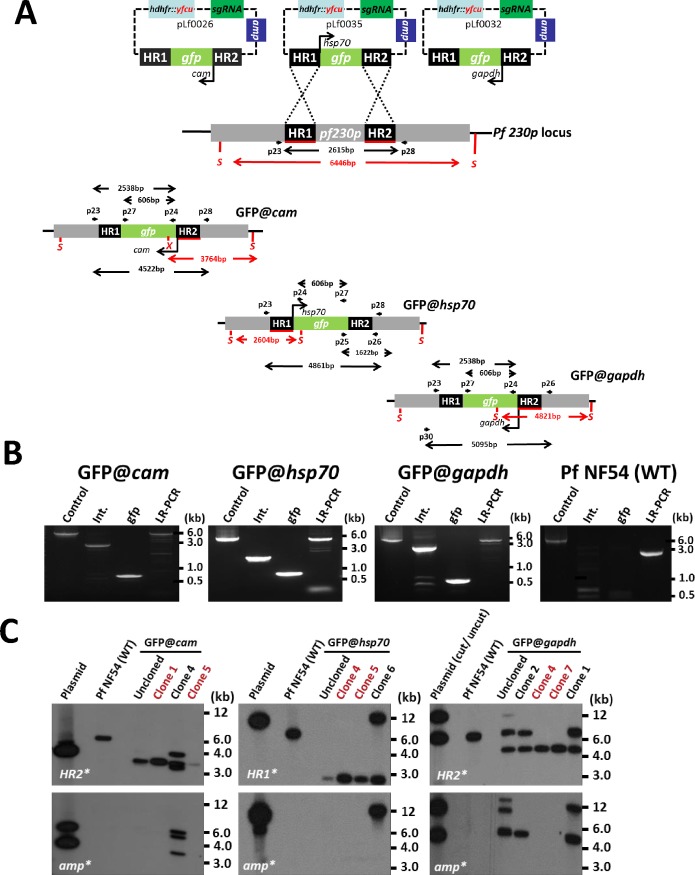
Generation of three *P*. *falciparum* reporter lines (GFP@*cam*, GFP@*hsp70*, GFP@*gapdh*) expressing GFP under control of different promoters. A. Schematic of the different sgRNA/donor constructs generated to introduce the GFP expression cassettes into the *P*. *falciparum* (*Pf*) *230p* gene locus. *Pf230p* homology regions (HR1, HR2) used to introduce the donor DNA (i.e. *gfp* expression cassettes), location of primers (p) and sizes of restriction fragments (S: *Spe*I, X: *Xho*I; in red) and PCR amplicons (in black) are indicated. Primer sequences (shown in black and bold) are shown in S1 Table. Note that the GFP expression cassette from GFP@*cam* and GFP@*gapdh* was cloned in the same orientation whereas that the GFP expression cassette form GFP@*hsp70* was cloned in the reverse orientation. See Fig 1 and S1 Fig. for details of the drug selectable marker and sgRNA sequences. This Figure is not shown to scale. B. Diagnostic (first 3 lanes) and long-range (LR-) PCR confirming correct integration of the GFP-expression cassettes into the *Pf230p* locus. Integration PCR of cloned parasites of GFP@*cam* (clone 1; primers p23/p24; expected size: 2538bp), GFP@*hsp70* (clone 5; primers p25/p26; expected size: 1622bp) and GFP@*gapdh* (clone 7; primers p23/p24; expected size: 2538bp). LR-PCR: GFP@*cam* (primers p23/p28; expected size: 4522bp), GFP@*hsp70* (primers p23/28; expected size: 4861bp) and GFP@*gapdh* (primers p30/p26; expected size: 5095bp); size of expected products shown in black and in bold in Fig 2A. Control PCR: *P*. *falciparum lisp2* gene (primers p21/p22; expected size: 5383bp); GFP: *gfp* gene (primers p24/p27; expected size: 606bp). C. Diagnostic Southern analysis confirms correct integration of the GFP-expression cassettes in the cloned lines of GFP@*cam*, GFP@*hsp70* and GFP@*gapdh*. *P*. *falciparum* NF54 (wild type WT) DNA, transfected parasite DNA after positive and negative selection (Uncloned; see Fig 1) and DNA from the different cloned lines was digested with *Spe*I and/or *Xho*I. The digested DNA fragments hybridized to probes recognizing either HR1 (GFP@*hsp70;* expected size: 2604bp) or HR2 (GFP@*cam;* expected size: 3764bp and GFP@*gapdh;* expected size: 4821bp) of the *Pf230p* target locus. In red are indicated the clones that have the correct genotype; absence of both plasmid and WT DNA (clone 1 and 5 for *GFP@cam;* clone 4 and 5 for *GFP@hsp70;* and clone 4 and 7 for *GFP@gapdh)*. As controls sgRNA/donor plasmid (Plasmid) DNA was digested and hybridised with a probe recognizing *ampicillin* (amp) of the donor DNA plasmid; *indicates probe used.

A 20 nucleotide crRNA sequence specific for *Pf230p* was identified using Protospacer software and this crRNA was introduced into the pLf0022 by replacing the *Btg*ZI adaptor sequence, resulting in a *Pf230p* sgRNA (sgRNA2) in the construct. This modified pLf0022 vector containing two HR of *Pf230p* and sgRNA2 created the plasmid pLf0024. We identified promoters to drive strong GFP transgene expression in blood stages. Specifically, promoters of three strong constitutively expressed genes based on published transcriptional profiles (RNAseq) data available from the PlasmoDB database (www.plasmodb.org). Genes were selected that had transcript levels (RNAseq values) that were similar or higher than that of the constitutively expressed *elongation factor 1α* (PF3D7_1357100), the promoter of this gene has been previously used in both *P*. *falciparum* and rodent models of malaria to drive the expression of reporter proteins [30]. These promoters were from the following genes: *calmodulin* (PF3D7_1434200; *cam*), *glyceraldehyde-3-phosphate dehydrogenase* (PF3D7_1462800; *gapdh*) and *heat shock protein 70* (PF3D7_0818900; *hsp70*); see S2 Table for data on the transcript levels of these genes. In the *cam* and *gapdh* promoter GFP-expression cassettes the *gfp* gene was placed under control of the *P*. *berghei calmodulin* transcriptional terminator (3’ UTR), whereas for *hsp70* the 3’UTR of *P*. *falciparum histidine-rich protein II* was used. In the final constructs the GFP expression cassette of *gfp*@*cam* and *gfp*@*gapdh* are consequently in a different orientation to *gfp*@*hsp70*; see the Material and Methods section for further details. Cloning of the different GFP expression cassettes in pLf0024 resulted in the following constructs; *gfp@cam* (pLf0026), *gfp@gapdh* (pLf0032) and *gfp@hsp70* (pLf0035), (Fig 2A and S2 Fig.).

### Generation of three transgenic reporter *P*. *falciparum* lines expressing GFP under different promoters

All construct were used to transfect *P*. *falciparum* ring stage parasites that were obtained from cultures after sorbitol synchronization. In each transfection 300 μl of pelleted infected RBC from cultures with a 6–15% parasitemia were mixed with 50 μg of the Cas9 and 50 μg of the donor plasmid. After transfection parasites were cultured in 10 ml flasks of an semi-automated culture system [31]. Twenty-four hours after transfection we applied ‘double’ positive selection by adding the drugs WR99210 and BSD to the cultures to select only for parasites that contain both plasmids. Drug pressure was maintained until infected RBC were detected by thin blood-smears analysis (usually 3 weeks after transfection). Subsequently, both drugs were removed from the cultures for 2–4 days, followed by the application of negative drug selection by the addition of 5-FC. This was performed in order to remove parasites that still retain episomal donor construct plasmid DNA, thereby enriching for transfected parasites that have the donor DNA integrated into their genome. To avoid any potential bystander killing effect of 5-FC at higher parasitemias we treated the cultures with 5-FC only after reducing the culture parasitemia to 0.5%. Negative drug pressure was maintained until thin blood-smears were parasite-positive (usually 7 days after application of 5-FC; Fig 1).

During both the positive and negative drug selection, parasites were analysed for GFP expression by fluorescence microscopy to determine the ratio of wild type and transgenic parasites present in the population. In multiple transfection experiments (exp.) with the three constructs with the different GFP-expression cassettes we obtained GFP-positive parasites after positive and negative selection (exp. 22 and 33 for pLf0026; exp. 44 for pLf0032 and exp. 35 for pLf0035). After negative selection the ratio of GFP+/GFP- parasites was determined and in the positive experiments the percentage of GFP-positive parasites ranged between 70 and 90%. Diagnostic PCR analysis for double cross-over integration after negative selection confirmed the presence of parasites with correct integration of the donor DNA. Based on the high GFP+/GFP- ratios and positive diagnostic PCR we proceeded to clone parasites from the following transfections exp. 22 (GFP*@cam*), exp. 35 (GFP*@hsp70*) and exp. 44 (GFP*@gapdh*). Cloning was performed by limiting dilution and GFP-positive clones were cultured in the semi-automated culture system for further genotyping by diagnostic PCR and Southern analysis. Both analyses confirmed correct integration of the donor DNA into the genome of the three cloned transgenic lines GFP*@cam* (exp. 22 clone 1 and 6), GFP*@hsp70* (exp. 35 clone 4 and 5) and GFP*@gapdh* (exp. 44 clone 4 and 7) and absence of the wild type DNA (Fig 2B and 2C). The cloning experiments indicated that >50% of the cloned lines we generated had the desired integration (i.e. GFP@*cam* 66%, 3 clones analysed; GFP@*gapdh* 57%, 7 clones analysed; and GFP@*hsp70* 66%, 3 clones analysed) (Fig 2C). In these 3 independent transfections the time from transfection to obtaining the marker-free GFP-expressing clones ranged between 10 and 12 weeks.

### *P*. *falciparum* reporter lines retain WT-like growth kinetics and drug sensitivity during blood stage development

The three reporter lines are free of a drug-selectable marker and consequently are easier to further genetically modify. In these reporter lines, using the same constructs described above, it is possible to delete, mutate or tag *P*. *falciparum* proteins in order to investigate their function and importance during parasite development. A prerequisite for additional genetic modifications using DNA constructs described above is that the parasite retains the same sensitivity to the drugs used to select parasite after transfection. It has been reported that parasites can spontaneously acquire blasticidin resistance when exposed to sustained BSD treatment independent of the *bsd* selectable marker [32]. We therefore compared the sensitivity of the three parasite lines to BSD and WR99210.

**Fig 3:**
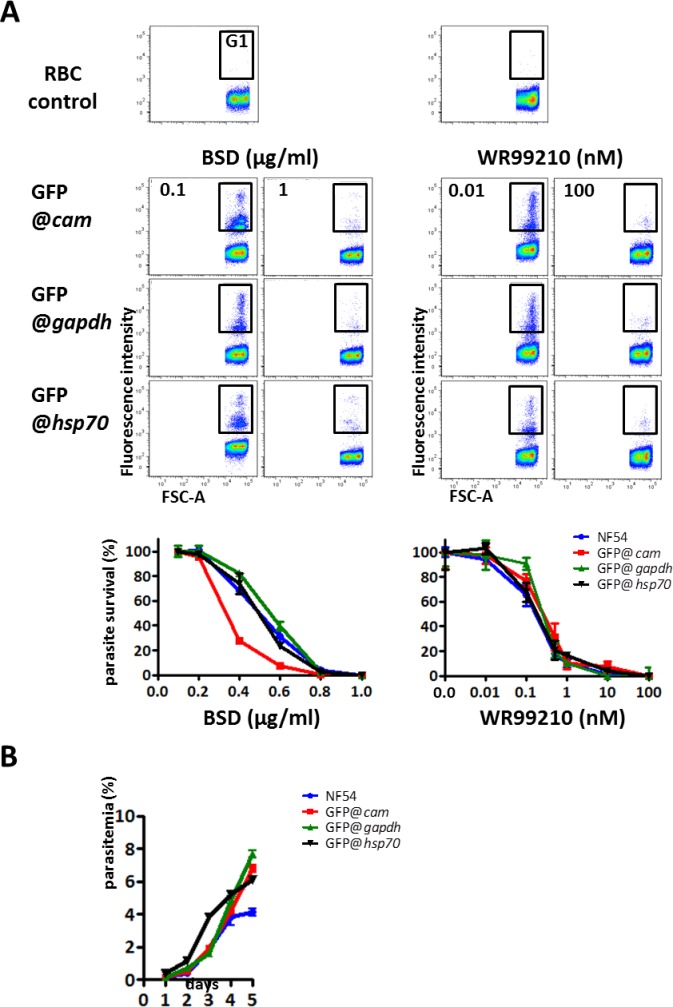
Drug-sensitivity and growth rate of asexual blood stages of three *P*. *falciparum* reporter lines (GFP@*cam*, GFP@*gapdh*, GFP@*hsp70*). A. Sensitivity to the drugs BSD and WR99210 as determined by flow cytometry in standard 72 h cultures in 96 well plates. Cultures of infected red blood cells (RBC) were incubated with different drug concentrations (in triplicate) and after 72 h samples were stained with the DNA-specific dye, Hoechst 33258, to determine parasitemia (% of infected RBC) by flow cytometry. Dot plots are shown of uninfected RBC (control, upper panel) selected using Forward Scatter parameter (FSC-A) and from cultures with the lowest and highest drug concentration (G1: infected RBC). Parasite survival is defined as the percentage of infected RBC in drug-treated wells divided by the percentage of infected RBC in non-treated wells multiplied by 100. IC_50_ values WR99210 (nM): (NF54 *Pf*WT) 0.16; (GFP@*cam*) 0.25; (GFP@*gapdh*) 0.27; (GFP@*hsp70*) 0.19. IC_50_ values BSD (μg/ml): (NF54 *Pf*WT) 0.48; (GFP@*cam*) 0.34; (GFP@*gapdh*) 0.54; (GFP@*hsp70*) 0.48. B. The growth rate of asexual blood stages in cultures maintained in the semi-automated culture system for a period of 5 days. Cultures were initiated with a parasitemia of 0.5%.

The drug-sensitivity of the asexual blood stages of clones of the three transgenic lines was determined in standard 72 h short-term culture assays in 96-wells culture plates. Serial dilutions of BSD and WR99210 were made with concentrations ranging from 0.1 to 1 μg/ml or 0.01 to 100 nM, respectively. Parasitemias in the culture wells were determined by flow cytometry and the parasite survival rate calculated (Fig 3A). The drug-sensitivity curves of the three reporter lines are comparable to that of wildtype NF54 parasites, with IC50 values between 0.34 and 0.54 μg/ml for BSD and 0.16 and 0.27 nM for WR99210 (Fig 3A).

In addition, in order to use these reporter lines to analyse the effect of additional modifications and/or inhibitors on the growth characteristics of *P*. *falciparum* blood stages, it is important that these parasites retain growth and development kinetics of the parental NF54 strain. We therefore compared the growth rate of the three different reporter lines. The growth rate of asexual blood stages was monitored by flow cytometry of samples collected daily from cultures maintained in the semi-automated culture system during a period of 5 days. Growth rates of the transgenic lines were highly comparable to that of NF54 wild type parasites and parasitemias increased from 0.5 to 4–8% during the culture period (Fig 3B) also the number of merozoites per schizont in the reporter lines was also comparable to that of the parental *P*. *falciparum* (NF54) WT line.

### GFP Expression of GFP@cam, GFP@gapdh and GFP@hsp70 during Blood Stage Development *In Vitro*

**Fig 4:**
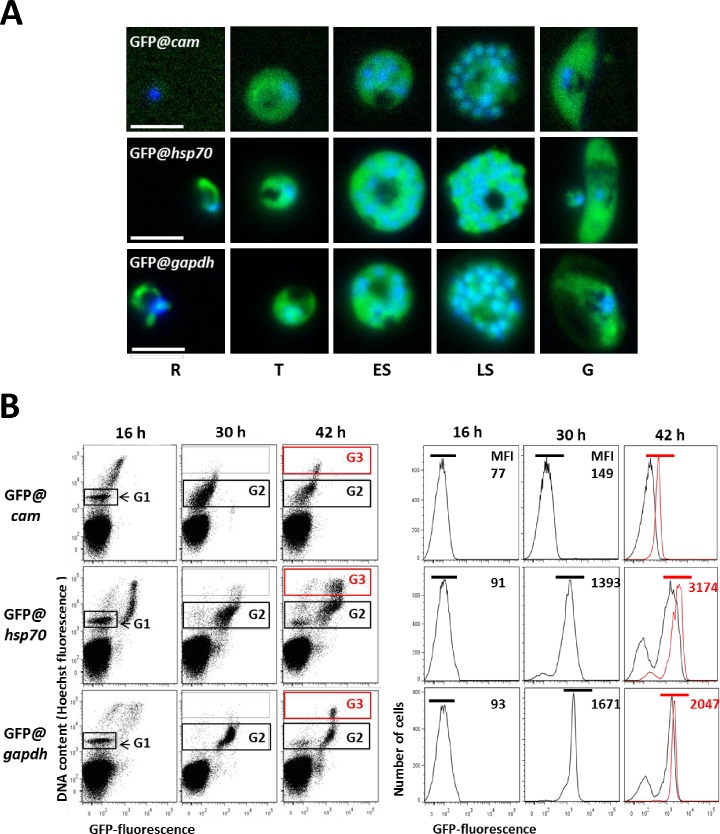
GFP-expression in blood stages of three reporter *P*. *falciparum* parasite lines (GFP@*cam*, GFP@*gapdh*, GFP@*hsp70*). A. Fluorescence microscopy of different blood stages. R: rings; T: trophozoites; ES: early schizonts; LS: late schizonts; G: gametocytes. Nuclei were stained with the DNA-specific dye Hoechst 33342. All pictures were recorded with standardized exposure/gain times to visualize differences in fluorescence intensity (GFP 0.7 s; Hoechst 0.136 s; bright field 0.62 s (1x gain)). In S3–S5 Figs. the complete set of microscope images are shown. B. Fluorescence intensity of rings (16 h), (late) trophozoites (30 h) and schizonts (42 h) as determined by flow cytometry. Infected red blood cells (RBC) were stained with the DNA-specific dye Hoechst 33258 to distinguish infected RBC from uninfected RBC and rings (Gate G1), trophozoites (Gate G2) from schizonts (Gate G3). Left side panels show dot plots of both Hoechst fluorescence intensity (DNA content) and GFP fluorescence intensity. Right side panels show GFP fluorescence intensity from parasites with either G1 and G2 (black) gate or G3 (red). MFI: mean fluorescence intensity and the black- (rings and trophozoites) and red- (schizonts) bar show the region selected to calculate the MFI.

We examined GFP expression in the different reporter lines during blood stage development by fluorescence microscopy. Expression of GFP was detectable in merozoites and ring forms of the GFP@*hsp70* and GFP@*gapdh* lines whereas all lines exhibited GFP expression in schizonts and gametocytes (Fig 4A and S3–S5 Figs.). This is in agreement with data on transcription of the three genes from which the promoters were used (S2 Table). Next we more precisely compared the GFP-fluorescence intensity of the different lines by examining the following synchronized stages by flow cytometry: rings (16 hours post invasion; hpi), trophozoites (30 hpi) and schizonts (42 hpi) (Fig 4B). Rings (G1), trophozoites (G2) and schizonts (G3) were distinguished based on their DNA content after staining with the DNA-specific dye Hoechst33258. While fluorescence increased during growth of trophozoites of all three lines, the fluorescence intensity did not further increase during schizogony. The GFP*@cam* parasites exhibited the lowest GFP expression with a mean fluorescence intensity (MFI) in trophozoites (MFI 149) only slightly higher that of uninfected cells whereas trophozoites of GFP*@hsp70* parasites and GFP@*gapdh* exhibiting much stronger GFP expression (MFI of 1339 and 1671, respectively). GFP*@hsp70* schizonts showed highest levels of GFP expression (MFI of 3174; Fig 4B).

## Discussion

Here we report the generation of *P*. *falciparum* reporter parasites expressing GFP under control of three different *P*. *falciparum* gene promoters using optimized CRISPR/Cas9 constructs and selection protocol. The introduction of CRISPR/Cas9 based genome editing to *P*. *falciparum* research has provided a powerful tool, which can be used to better and faster interrogate parasite gene regulation and function [33]. Before CRISPR/Cas9, modifications of the *P*. *falciparum* genome, such as gene disruption or mutation or the introduction of transgenes into the genome, required 1–3 months of continuous culture to select for parasites in which episomally maintained plasmids became integrated into the parasite’s genome, either by single or by double cross-over recombination [33]. Further, the process of generating cloned genetically modified and drug-selectable marker-free parasites would typically take 5 months or more to complete [20]. With the methods described here we are able to generate cloned marker-free parasite lines that stably express reporter proteins within a period of 10–12 weeks.

The constructs we have generated can be modified and used in future studies as template constructs to remove or introduce transgenes into the *P*. *falciparum* genome, for example they could be used to generate reporter lines that express additional (fluorescent/bioluminescent) reporter proteins or used to analyse the regulatory elements that control *Plasmodium* gene expression.

In addition the sgRNA/donor DNA construct can be adapted to permit the introduction of transgenes into other *P*. *falciparum* genetic loci by adapting the homology regions in the donor plasmid, or the construct can be modified to introduce other transgene (e.g. reporter) expression cassettes under the control of a variety of regulatory promoter and transcription terminator (5’- and 3’- UTR) regions. All the plasmids and *P*. *falciparum* mutant lines described in this study are available on request.

In the sgRNA/donor plasmid we have placed the selectable marker cassette outside the donor DNA cassette which then does not result in the introduction of the selectable marker into the parasite genome upon repair of the target locus. Hence the locus can be modified without the inclusion of a selectable marker cassette. The same type of constructs can also be used to perform other genetic manipulations, notably gene-disruption and gene-mutations, which can be used to interrogate gene function and importance. As a consequence of the absence of a drug-selectable marker in the genome of the mutants, these constructs can be adapted and used to create rapid successive genetic modifications in the same parasite line. For example, ‘doubly’ modified parasites that contain both a reporter gene as well as a disrupted (or mutated) gene. Such rodent malaria mutants have been used extensively to analyse the phenotypic consequences of a gene disruption/mutation using a variety of imaging technologies. Moreover, our transfection protocol permits for a more rapid generation of multiply modified parasites since it is possible to further transfect the uncloned population of a modified parasite (after parasite emerge from negative selection). With the high transfection efficiencies we observe in our transfection protocol, combined with the rounds of positive and negative selection, it is possible to create cloned and SM free, doubly transgenic parasites in the same time (~5 months) it would have taken to create a single SM-free genetic modification in *P*. *falciparum* using conventional approaches. Indeed a cloned *P*. *falciparum* double gene deletion mutant (PfΔ*mrp1*Δ*mrp2*), which still contained a drug-selectable marker, has been recently reported and it required 3 independent transfections, 2 rounds of cloning, 1 round of SM recycling and took nearly a year to generate [34].

To select for parasites with the donor DNA integrated into the genome we made use of a selectable marker cassette containing a fusion gene of the human dihydrofolate reductase (h*dhfr*) positive selectable marker and a negative selectable marker, the bifunctional protein that combines yeast cytosine deaminase and uridyl phosphoribosyl transferase (y*fcu*). Negative selection based on yFCU expression has been used for both for genetic modification of *P*. *falciparum* [35] and for rodent malaria parasites [36]. The drug 5-FC efficiently kills parasites that express *y*FCU both in *in vitro* cultures and *in vivo* in laboratory animals. We established that the positive/negative selectable marker (h*dhfr*::y*fcu*) cassette was functional in *P*. *falciparum* by transiently transfecting parasites with a plasmid (pLf0033) containing the h*dhfr*::y*fcu* fusion cassette and obtaining parasites using positive drug (WR99210) selection, followed by the application of negative drug (5-FC) selection that killed the parasites demonstrating that they were sensitive to this drug. In the CRISPR/Cas9 method described here, we apply negative selection to kill parasites that still retain the sgRNA/donor plasmid DNA, enriching for the population of parasites where the donor DNA has integrated into the parasite’s genome. Negative selection will also not kill ‘wild type’ parasites that are still present in the population, i.e. parasites that had lost the donor DNA construct without an integration event. From the ratio of GFP-positive and GFP-negative parasites present in the cultures after negative selection, as well as the results of cloning, we found that percentage of stable transgenic parasites was in excess of 50% and therefore the presence of wild type parasites appeared not to be an obstacle for obtaining the desired mutants. It has also been reported that homologous repair mediated by a donor DNA in *P*. *falciparum* after CRISPR/Cas9 transfection appears to more efficient than parasite mediated non-homologous end joining repair of DNA, which therefore favours the selection of transgenic parasites over either wild type parasites or parasites that have repaired the double strand break through the introduction of a site specific frame shift [26]. Though it was not necessary in this study, fluorescent reporter parasites can be further enriched or indeed cloned after negative selection by flow/FACS sorting of fluorescent cells.

We generated in this study three different reporter parasite lines principally to select for constitutive and strong promoters that can be used to drive reporter gene expression. In previous studies only a limited number of promoters have been used for genetic modification of *P*. *falciparum*. For driving transgene expression *eef1α* [17, 18] and *hsp86* [37, 38] have been reported, while *cam* and *hsp86* have been used to drive expression of selectable markers [39]. By comparing RNAseq data of blood stages we selected three genes with expression levels higher than *eef1α*, a promoter that has been previously used to generate GFP-expressing reporter *P*. *falciparum* lines [17, 18]. We found that the *cam* promoter resulted in relatively weak GFP-expression compared to *gapdh* and *hsp70* which is in agreement with the RNAseq data. GAPDH is an enzyme involved in glycolysis, the main pathway for ATP production in *Plasmodium* [40] and therefore this protein is likely to be expressed throughout the complete life cycle. Indeed proteome analyses of oocysts and sporozoites provide evidence for high abundance of GAPDH in these stages (PlasmoDB; www.plasmodb.org). Therefore, we believe, that this promoter can be a useful tool to drive transgene expression throughout the complete life cycle.

Currently several CRISPR/Cas9 methods using two plasmid based strategies have been described for *P*. *falciparum* genetic modification. The constructs described in Ghorbal *et al*. [22] like our study, have both the sgRNA and donor sequences on one plasmid. In contrast to our method, the use of these constructs result in generation of mutants, both deletion mutants and transgenics, that carry a drug selectable marker into their genome. This method has the advantage that there is no need for negative selection to remove parasites that retain the plasmid. While in the Ghorbal *et al*. study marker free point mutation mutants have been generated, it is unclear if their constructs could be used for complete gene deletion or a large genetic insertion without inclusion of a drug selectable marker. As both our and the Ghorbal *et al*. method have the *cas9* gene introduced on a separate plasmid, only one plasmid has to be modified for each subsequent modification (i.e. changing the sgRNA and/or donor sequences) and therefore multiple *P*. *falciparum* genes can be targeted using a pool of constructs in a single transfection experiment. Consequently, multiple mutants can be obtained from a single transfection experiment. In contrast the method described by Lu *et al*. [26], where the sgRNA and donor sequences are on separate plasmids, requires both plasmids to be modified to create an additional gene modification/disruption. However the strategy described by Lu et al, where the donor DNA is on a separate plasmid, allows for the introduction of larger DNA inserts into the parasite’s genome.

When we examined clones from the 3 different transgene mutants 57–66% had the expected genotype. The other clones were WT clones or, more commonly, clones where the donor construct appears to integrate into the parasite genome by single cross-over recombination. While ‘single cross-over’ parasites retain the h*dhfr*::y*fcu* SM a reduction in sensitivity to 5-FC is expected, due to reduction of h*dhfr*::y*fcu* copies in the genome compared to parasites containing multiple episomal plasmids. A reduction in the selection of the clones with the undesired genotype can be accomplished by increasing the 5-FC concentration during selection and/or by increasing the expression of the h*dhfr*::y*fcu* SM, for example by replacing the promoter of the SM (*Pfhsp86*) with that of a more highly expressed gene (e.g. *Pfhsp70*). This is particularly important when a gene deletion may result in a growth defect (unlike *Pf230p*) and WT parasites may ‘over grow’ the deletion mutant.

In summary, we have created improved constructs and describe an efficient transfection protocol to create modified *P*. *falciparum* parasites and these reporter parasites are suitable for further genetic modifications since they are SM-free. Improving the ability to perform genetic manipulations, including making it easier to perform successive gene-deletions and gene-mutations, will not only be of value to interrogate parasite gene function but also for the development of multiple attenuated malaria parasites suitable for vaccination [41].

## Materials and Methods

### Parasites and *in vitro* cultivation of blood stages

*P*. *falciparum* parasites from the NF54 strain [42] were obtained from the Radboud University Medical Center (Nijmegen, The Netherlands). These parasites were used to generate the different transgenic parasite lines. Parasites were cultured following the standard conditions in RPMI-1640 culture medium supplemented with L-Glutamine and 25mM HEPES (Gibco Life Technologies) to which was added 50 mg/L hypoxanthine (Sigma). Culture medium was supplemented with 10% human serum and 0.225% NaHCO_3_. Parasites were cultured at a 5% hematocrit under 4% O_2_, 3% CO_2_ and 93% N_2_ gas-conditions at 75 rpm at 37°C in a semi-automated culture system in 10ml flasks (Infers HT Multitron and Watson Marlow 520U)[22]. Fresh human serum and human red blood cells (RBC) were obtained from the Dutch National Blood Bank (Sanquin Amsterdam, the Netherlands; permission granted from donors for the use of blood products for malaria research and microbiology test for safety). RBC of different donors were pooled every two weeks, washed twice in serum free RPMI-1640 and resuspended in complete culture medium to 50% haematocrit. Human serum of different donors were pooled every 4–6 months and stored at -20°C until required.

### Generation of new standard CRISPR/Cas9 constructs

The first Cas9-expressing construct (Cas9; pLf0019), was generated by replacing the drug-selectable marker y*dhodh* of the standard construct pUF1-Cas9 [22] by the *bsd* selectable marker obtained from the pMV-FLPe construct [20] using the restriction enzymes *EcoR*I/*Spe*I. The second construct, containing both the sgRNA as well as the donor DNA sequences (sgRNA/Donor construct; pLf0022), was generated in multiple cloning steps. This construct contains both the sgRNA expression cassette and the selectable marker cassette containing the fusion gene of the positive selectable marker *hdhfr* and the negative selectable marker y*fcu* (yeast cytosine deaminase/uridyl phosphoribosyl transferase [28]. Briefly, the sgRNA-expression cassette under control of the *Plasmodium u6* RNA promoter (PF3D7_1341100) containing the BtgZI adaptor sequence was digested from pL6-eGFP[22] using the restriction enzymes *Nco*I/*Aat*II and cloned in the intermediate plasmid pLf0051. The *P*. *falciparum hsp86* promoter (PF3D7_0708400) was obtained from JCK-3 plasmid (obtained from Prof. R.W. Sauerwein, Nijmegen, the Netherlands) using the restriction enzymes *Pvu*II/*SexA*I and was cloned into the *P*. *berghei* transfection construct pL0034 (RMgm-687; www.pberghei.eu) resulting in plasmid pLf0033. The *P*. *falciparum hsp86* promoter, replaced the existing *P*. *berghei eef1α* promoter and was placed upstream of the h*dhfr*:: y*fcu* fusion gene (positive/negative selectable marker) and the *P*. *berghei dhfr/ts* (PBANKA_0719300) transcriptional termination (3’UTR) sequence, which were already present in pL0034 [28]. Subsequently the complete cassette was digested from pLf0033 with the *Stu*I/*Kpn*I restriction sites and cloned into pLf0051 with *EcoR*V/*Kpn*I, resulting in the final construct pLf0022. This construct contains additional restriction sites for introducing homology/targeting sequences to target any gene of interest such as *Stu*I/*Sac*II and *ApaI*/*Hind*III (see below).

### *P*. *falciparum* 230p (*Pf230p*) targeting constructs

Constructs were designed to target the *Pf230p* locus (PF3D7_0208900) in the *P*. *falciparum* genome. To generate the *Pf230p* targeting vectors, plasmid pLf0022 (see above) was modified introducing two homology regions targeting *Pf230p*. Homology region 1 (HR1) was amplified using primers P1/P2 and homology region 2 (HR2) with P3/P4 from *P*. *falciparum* NF54 genomic DNA (see S1 Table for primer details). HR1 was cloned in pLf0022 using restriction sites *Stu*I/*Sac*II and HR2 using *ApaI*/*Hind*III, resulting in intermediate plasmids CM162 and CM163. A guide sgRNA (sgRNA2) sequence for *Pf230p* was identified using the Protospacer software (alpha version; https://sourceforge.net/projects/protospacerwb/files/Release/) and was amplified using the primers P7/P8. This sgRNA was selected based on the best off targets hits score throughout the genome given by Protospacer and the total number of mismatches of the sgRNA with respect to the PAM site. A 20 bp guide sgRNA, surrounded by 15 bp vector specific DNA necessary for InFusion cloning (HD Cloning Kit; Clontech), was annealed and used to replace the BtgZI adaptor as previously described [22], resulting in construct pLf0024. The construct was digested with *Bln*I and *Nru*I to evaluate the successful cloning of the sgRNA and later confirmed by Sanger sequencing using primers P9/P10.

The generation of the three *Pf230p* targeting constructs that contain the *gfp* gene under different promoters were constructed in multiple cloning steps. The promoters were selected based on published transcript levels of their genes in asexual blood stages (RNA seq data available in PlasmoDB, www.plasmodb.org). The promoters of the following genes were selected *cam* (*calmodulin*; PF3D7_1434200); *gapdh* (*glyceraldehyde-3-phosphate dehydrogenase*; PF3D7_1462800) and *hsp70* (*heat shock protein 70*; PF3D7_0818900). The *cam* promoter was amplified from NF54 genomic DNA using primers P11/P12 and cloned in the intermediate plasmid pLf0052 using the enzymes *Aat*II/*Bam*HI. This plasmid contains the *gfp* expression cassette with the *P*. *falciparum cam* promoter region and the 3’ UTR region from the *calmodulin* gene from *P*. *berghei* ANKA (PBANKA_1010600), which was previously amplified from intermediate plasmid Plf0012 using primers P17/P18. The *gfp@cam* expression cassette was obtained by digestion with *Apa*I/*Pvu*II and cloned into plasmid pLf0024 (see above) using restriction sites *Apa*I/*EcoR*V, resulting in the final *gfp@cam* construct pLf0026.

The *gapdh* promoter was amplified from NF54 genomic DNA using primers P13/P14 and used to replace the *cam* promoter by the *gapdh* promoter in intermediate plasmid pLf0052 using the restriction sites *Aat*II/*BamH*I. The complete *gfp@gapdh* expression cassette from this plasmid was digested with *Apa*I/*Pvu*II and cloned into pLf0024 (see above) using restriction sites *Apa*I/*EcoR*V, resulting in the final *gfp@gapdh* construct pLf0032.

The complete *gfp@hsp70* expression cassette was obtained by digestion from the intermediate plasmid pLf0053 using restriction enzymes *Apa*I/*Pvu*II and cloned into pLf0024 (see above) using restriction sites *Apa*I/*EcoR*V resulting in the final *gfp@hsp70* construct pLf0035. The *hsp70* promoter was amplified from NF54 genomic DNA using primers P15/P16. For the 3’UTR of the *gfp@hsp70* expression cassette the 3’UTR of the gene encoding the *histidine-rich protein II* (PF3D7_0831800) was amplified with primers P19/P20 from the plasmid pHHT-FRT-(GFP)-Pf52 [20].

The *gfp@cam* and *gfp@gapdh* plasmids were created using the intermediate plasmid pLf0052, resulting in the same orientation of the GFP expression cassette and the same 3’ UTR (*calmodulin* gene) whereas the *gfp@hsp70* plasmid was created using an intermediate plasmid pLf0053 which resulted in the reporter cassette in a reverse orientation and the *histidine-rich protein II* 3’UTR.

All PCR amplifications were performed with high-fidelity Phusion DNA polymerase (New England Biolab) following the recommended protocols, except for the promoters (*cam*, *gapdh* and *hsp70*) that were amplified with KOD Hot Start polymerase (Novagen) under standard conditions. All cloning and plasmid amplifications were done in *Escherichia coli*, XL10-Gold Ultracompetent Cells (Stratagene). Details of the primer sequences are shown in S1 Table.

### Transfection and selection of transgenic parasites

Plasmids for transfection were isolated from 250 ml cultures of *Escherichia coli*, XL10-Gold Ultracompetent Cells (Stratagene) by maxi-pep (using HiSpeed® Plasmid Maxi Kit (Qiagen®)) to generate the 50 μg of DNA used per transfection. Transfections were performed using ring stage parasites obtained from cultures with a parasitemia of 6–15% that were synchronized by 5% D-sorbitol treatment 2 days before transfection [43]. Infected RBC were pelleted by centrifugation (1150*g*, 5 min.) and 300 μl of the pelleted cells were transferred to a 0.2 cm cuvette and mixed with ~50 μg of each circular plasmid (Cas9 construct and sgRNA/Donor construct) in 100 μl cytomix [44]. Electroporation was performed with a single pulse (310 V and 950μF) in the Biorad Gene Pulser Xcell electroporator (including CE- and PC module). After transfection cells were immediately transferred in a 10 ml culture flask and cultures were maintained under standard conditions in the semi-automated culture system (see above).

Selection of transfected parasites was performed by applying ‘double’ positive selection 24 h after transfection using the drugs WR99210 (2.6 nM) and BSD (5 μg/ml). For WR99210 100 μl of a stock solution (2.6 μM) was added to 100 ml complete culture medium resulting in a final concentration of 2.6 nM. To prepare the WR99210 stock-solution WR99210 was dissolved in DMSO (100mM). For BSD 50 μl of a stock solution (10mg/ml) was added to 100 ml complete culture medium resulting in a 5 μg/ml final concentration. The drug pressure was maintained until thin blood-smears were parasite-positive (usually after 14 to 26 days). Positive selection will select for the parasites that were transfected successfully with both plasmids (Cas9 and sgRNA/Donor constructs). Subsequently, both drugs were removed from the cultures for 2–4 days, followed by applying negative selection by addition of 5-Fluorocytosine (5-FC; 130 μl of a stock solution (0.77 mM) in 100 ml complete medium with a final concentration of 1 μM; [45]) in order to eliminate parasites that retained the sgRNA/Donor construct as episomal plasmid and enriching for those transfected parasites where the donor DNA had integrated into the genome. Negative drug pressure was maintained until thin blood-smears were parasite-positive (usually after 7 days). During both positive and negative selection period, parasites were analysed for GFP expression by fluorescence microscopy (see below) to determine the ratio of wild type and mutant parasites present in the population. After negative selection parasites were harvested from cultures with 4 to 10% of parasitemia for genotyping by diagnostic PCR and Southern analysis (see below).

### Cloning of transgenic parasites

Based on the percentage of GFP-positive parasites in cultures after negative selection and PCR confirmation of double cross-over integration the transgenic parasites were cloned by the method of limiting dilution as previously described [46] with minor modifications. Briefly, infected RBC from cultures with a 4% to 10% parasitemia were diluted with uninfected RBC to 10^5^ infected RBC/100 μl in 2 ml culture medium (1% hematocrit and 20% serum). Serial dilutions were then performed with uninfected RBC in complete medium (1% hematocrit and 20% serum) and cultured in a total volume of 100 μl incubated in 96 well plates, resulting in 8 rows with the following numbers of parasites per well in the different rows: 100, 10, 5, 2.5, 1.25, 0.6, 0.3, 0.15. Plates were incubated in a Candle Jar at 37°C and culture medium was changed every other day. Every 5 days RBC were added resulting in an increase of the hematocrit from 1% to 5%. Between days 10–14 samples were collected for thick smear analysis from the rows with the highest numbers of infected RBC/well; 50 μl medium was removed and from the remaining culture 5 μl was used directly for preparing thick smears. At day 21 thick smears were made from all rows. Clones were selected from dilutions/row with less than 30% of the wells parasite positive. These clones were transferred in 10 ml culture flasks at 5% hematocrit under standard culture conditions (see above) in the semi-automated culture system for collection of parasites for further genotype and phenotype analyses (see below).

### Genotype analysis of cloned transgenic parasite lines

For genotyping by diagnostic PCR and Southern analysis were performed from material isolated from infected RBC obtained from 10ml cultures (parasitemia 3–10%), pelleted by centrifugation (1150 *g*; 5 min.). RBC were then lysed with 5–10 ml of cold (4°C) erythrocyte lysis buffer (10x stock solution 1.5 M NH_4_Cl, 0.1 M KHCO_3_, 0.01 M Na_2_EDTA; pH 7.4; [43]) and parasites were treated with RNAse and proteinase-K before DNA isolation by standard phenol-chloroform methods. Correct integration of the donor construct was analysed by standard and long-range PCR (LR-PCR). In brief, for the GFP*@cam* and GFP*@hsp70* expression cassette integration was confirmed by LR-PCR using the primers P23/P28 and for GFP*@gapdh* the integration was confirmed using the P30/P26 primers (see S1 Table for details of the primers). The LR-PCR fragments were amplified using KOD Hot start polymerase following standard conditions with an annealing temperature of 53.5°C for 15 s and an elongation step of 68°C for 9 min. All other PCR settings were according to manufacturer's instructions.

Southern blot analysis was performed with genomic DNA digested with *Xho*I and/or *Spe*I restriction enzymes (4 h at 37°C) in order to confirm integration of the expression cassette into the *Pf230p* locus. Digested DNA was hybridized with probes targeting the *Pf230p* homology regions, amplified from NF54 genomic DNA by PCR using the primers P1/P2 for HR1 and P3/P4 for HR2 respectively.

### Phenotype analysis of parasites

The growth rate of asexual blood stages of clones of the three transgenic lines was monitored in 10 ml cultures maintained in the semi-automated culture system under standard culture conditions (see above). Briefly, a 0.5% parasitemia culture was established in complete culture medium at a haematocrit of 5%. Medium was changed twice daily and the culture maintained for a period of 5 days without refreshing RBC. For determination of the course of parasitemia, triplicate samples of 100 μl were collected daily from all cultures and cells pelleted by centrifugation (9485 *g*; 30s). The culture medium was then removed and cells were washed twice in 1X PBS before and after fixation with 0.25% glutaraldehyde (30 min. at 4°C). Fixed RBCs were stained with the DNA-specific dye Hoechst33258 in 1 ml of PBS by adding 4 μl of a 500 μM stock-solution (final concentration 2 μM). Samples were stained for 1hr at 37°C in the dark and analysed by FACS [47].Hoechst-fluorescence intensity of stained cells was measured using an LSRII flow cytometer (Becton Dickinson, Mountain View, CA, USA) and the data was analysed using FlowJo software (Treestar, Ashland, OR, USA). At least 50 000 cells were analysed per sample and the parasitemia was determined by FACS using an UV laser (355 nm) and band pass filter 450/50 nm [47] and examining the number of Hoechst-positive and Hoechst-negative cells. RBCs were selected by gating on Forward and Side Scatter parameters (FSC and SSC, respectively). Doublets are excluded by using FSC-Area and FSC-height parameters.

Drug-sensitivity of asexual blood stage parasites from cloned lines of the three transgenic lines was analysed as described previously [20], with the following modifications. Infected RBCs (0.1%-0.5% parasitemia) at 1% of haematocrit were cultured in 96-wells culture plates in a Candle Jar (in complete medium and 20% human serum). To each well containing 100 μl of the infected RBC culture was added another 100 μl of culture medium containing different concentrations of BSD or WR99210 with concentrations ranging from 0.1 to 1μg/ml BSD or from 0.01 to 100 nM WR99210; each drug concentration was performed in triplicate wells). Serial dilutions were made from stock-solutions of 1 mg/ml and 1 mM of BSD and WR99210, respectively. Medium of the cultures was changed daily.

Determination of the parasitemia in the culture wells was determined at 72 h after start of the cultures by flow cytometry. Briefly, cells were pelleted by centrifugation (9485 *g*, 30 s) and cells were washed twice in 1X PBS before and after fixation with 0.25% glutaraldehyde (30 min. at 4°C). Fixed RBC cells were stained with the DNA-specific dye Hoechst 33258 in 1 ml of PBS by adding 4 μl of a 500 μM stock-solution (final concentration 2 μM). Samples were stained for 1 h at 37°C in the dark and analysed by flow cytometry [47]. Determination of parasitemia (= percentage of infected RBC) by flow cytometry was determined as described above and was analysed using GraphPad Prism software (GraphPad software, Inc., US). Parasite survival is defined as the percentage of infected RBC in drug-treated wells divided by the percentage of infected RBC in non-treated wells multiplied by 100. For calculation of the survival curves, the mean fluorescence intensity value of samples with the highest drug concentration (i.e. with maximum inhibition of growth) is subtracted from the mean fluorescence intensity value of the samples with the other drug concentrations and the control samples without drug. The mean parasitemia of the control samples without drug is set at 100% and the mean parasitemia of the highest drug concentration is set at 0% for calculation of the parasite survival. Growth inhibitory curves and statistical analysis of the data is performed using the GraphPad Prism software. The non-linear regression function for sigmoidal dose-response (variable slope) of the GraphPad Prism software is used to calculate the (best-fit) EC_50_ values.

GFP expression in different blood stages was analysed by standard fluorescence microscopy. In brief samples of approximately 200 μl were collected from 10 ml infected cultures with parasitemias between 4 and 10%. The RBC samples were stained with the DNA-specific dye Hoechst 33342 by adding 4 μl of a 500 μM stock-solution to a final concentration of 10 μM for 20 min. at 37°C. Five μl of the preparation was mounted on a microscopic slide under a cover slip to visualize the parasites by Hoechst and GFP fluorescence using a Leica fluorescence MDR microscope (100x magnification). Pictures were recorded with a DC500 digital camera microscope using Leica LAS X software and with the following exposure times: GFP 0.7 s; Hoechst 0.136 s; bright field 0.62 s (1x gain).

The relative GFP-fluorescence intensity of different asexual blood stages was analysed by flow cytometry. Triplicate samples of 100 μl of infected RBC were collected from cultures that had been synchronized with sorbitol and cultured in the semi-automated *in vitro* system. Samples were collected at 30 and 42 h after synchronization and resuspended in 1 ml of culture medium containing 5% serum. Cells were stained with the DNA-specific dye Hoechst33258 by adding 20 μl of a 500 μM stock-solution to a final concentration of 10 μM [47]. Staining was performed for 30 min. at 37°C. GFP and Hoechst fluorescence intensity was determined using a LSRII flowcytometer (Becton Dickinson, Mountain View, CA, USA) and the data was analysed using FlowJo software (Treestar, Ashland, OR, USA). 100.000 cells were analysed per sample and RBC were selected by gating on FSC and SSC. Doublets are excluded by using FSC-Area and FSC-height parameters. Excitation of cells for Hoechst33258 was performed with a UV laser (355 nm) and band pass filter 450/50 nm and for GFP with a blue laser (488 nm) and a band pass filter of 530/30 nm. The GFP fluorescence intensity was determined of the haploid blood stages (rings and trophozoites; Gate 1) and polyploid blood stages (schizonts; Gate 2). Haploid and polyploid blood stages were distinguished based on Hoechst-fluorescence intensity [47]. Data generation was performed using the FACS DIVA software (Becton Dickinson) and analysed with FlowJo software.

## Supporting Information

S1 Fig Drug sensitivity of *P. falciparum* parasites expressing the *hdhfr*::*yfcu* fusion cassette. A. Vector map of pLf0033, expressing the h*dhfr*::y*fcu* SM cassette, used for transient transfection. B. Growth of NF54 blood stage parasites in the absence or presence of the positive drug, WR99210 (WR; 2.6nM final concentration). WT *P*. *falciparum* NF54 parasites (NF54) were episomally transfected with the plasmid pLf0033, encoding a positive/negative drug selection h*dhfr*::y*fcu* fusion cassette (NF54 ^+/-^plasmid) and selected under positive (WR) selection. Cultures were diluted to ~0.5% parasitemia with fresh erythrocytes when parasitemia reached 5–10%. C. Episomally transfected *P*. *falciparum* parasites (NF54 ^+/-^plasmid), which were initially selected under positive (WR) selection, and WT *P*. *falciparum* NF54 parasites were subjected to negative (5-FC 1μM final concentration). Cultures were diluted to ~0.5% with fresh erythrocytes when parasitemia reached 5–10%.
(TIFF)

S2 Fig Main Constructs used in this study. A. Basic constructs: pLf0019 for Cas9-expression construct with the *bsd* selectable marker; pLf0022 sgRNA/donor construct and pLf0024 for targeting the *Pf230p* locus. B. Constructs used for introduction of the GFP-expression cassettes into the *P*. *falciparum* genome: pLf0026 for *gfp@cam*, pLf0032 for *gfp@gapdh* and pLf0035 for *gfp@hsp70* into *Pf230p*.
(TIFF)

S3 Fig Fluorescence microscopy of GFP@*gapdh* blood stages parasites. R: rings; T: trophozoites; ES: early schizonts; LS: late schizonts; G: gametocytes. Nuclei were stained with the DNA-specific dye Hoechst 33342. All pictures were recorded with standardized exposure/gain times to visualize differences in fluorescence intensity (GFP 0.7 s; Hoechst 0.136 s; bright field 0.62 s (1x gain)).
(TIFF)

S4 Fig Fluorescence microscopy of GFP@*hsp70* blood stages parasites. R: rings; T: trophozoites; ES: early schizonts; LS: late schizonts; G: gametocytes. Nuclei were stained with the DNA-specific dye Hoechst 33342. All pictures were recorded with standardized exposure/gain times to visualize differences in fluorescence intensity (GFP 0.7 s; Hoechst 0.136 s; bright field 0.62 s (1x gain)).
(TIFF)

S5 Fig Fluorescence microscopy of GFP@*cam* blood stages parasites. R: rings; T: trophozoites; ES: early schizonts; LS: late schizonts; G: gametocytes. Nuclei were stained with the DNA-specific dye Hoechst33342. All pictures were recorded with standardized exposure/gain times to visualize differences in fluorescence intensity (GFP 0.7 s; Hoechst 0.136 s; bright field 0.62 s (1x gain)).
(TIFF)

S1 Table List of primers used in this study. (DOCX)

S2 Table Transcript abundance (RNAseq RPKM values) during asexual blood stage development of four genes. Data obtained from PlasmoDB (www.plasmodb.org; published in Otto *et al*. (2010) *Mol*. *Microbiol*. *76(1)*:*12–24*).
(DOCX)
