# Supplementary figures and images for: Rapid Generation of Marker-Free P. falciparum Fluorescent Reporter Lines Using Modified CRISPR/Cas9 Constructs and Selection Protocol
Source: PLoS One. 2016 Dec 20;11(12):e0168362. doi: 10.1371/journal.pone.0168362 (PMC5172577; doi:10.1371/journal.pone.0168362)

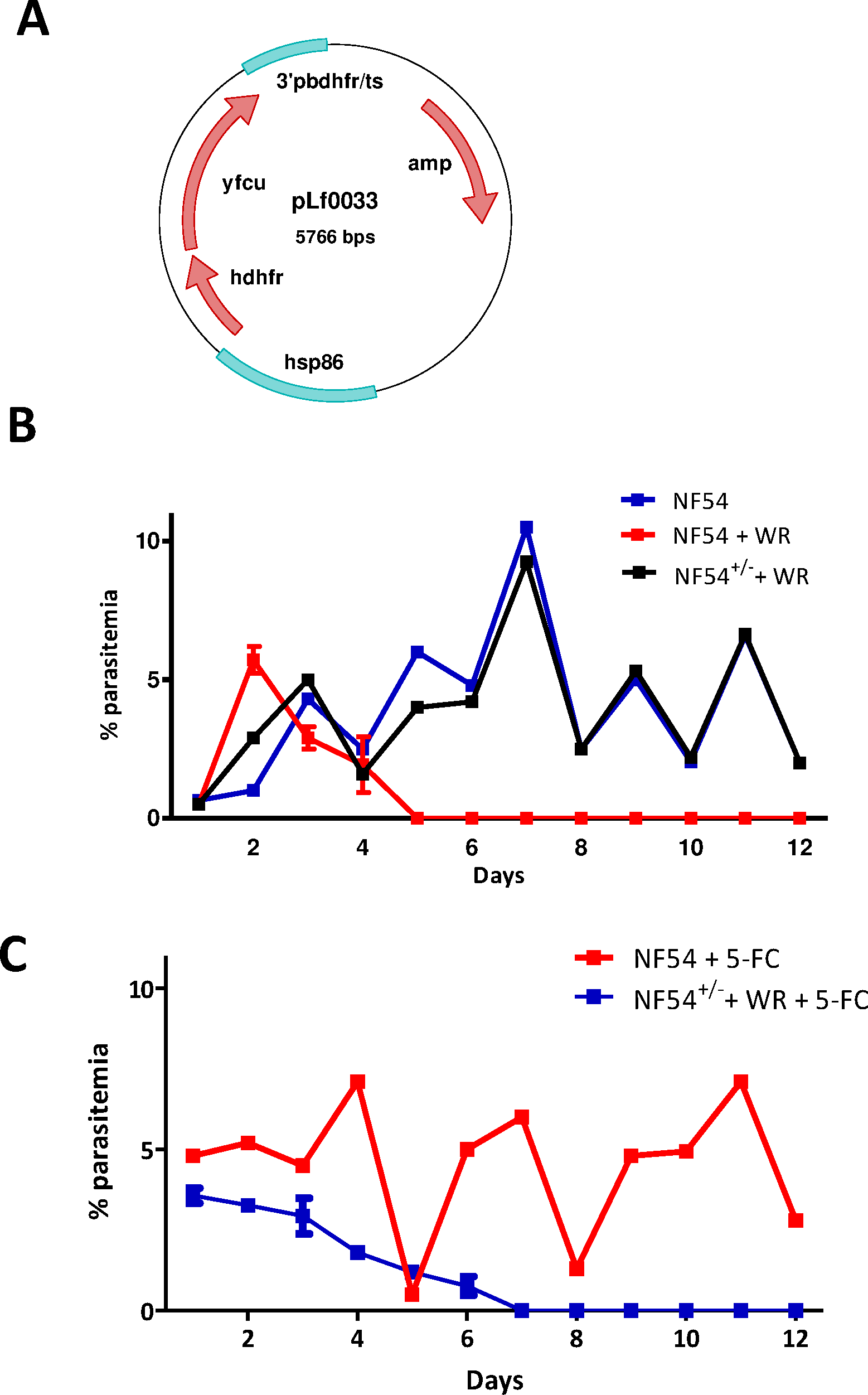

Supplement: S1 Fig — A. Vector map of pLf0033, expressing the hdhfr::yfcu SM cassette, used for transient transfection. B. Growth of NF54 blood stage parasites in the absence or presence of the positive drug, WR99210 (WR; 2.6nM final concentration). WT P. falciparum NF54 parasites (NF54) were episomally transfected with the plasmid pLf0033, encoding a positive/negative drug selection hdhfr::yfcu fusion cassette (NF54 +/-plasmid) and selected under positive (WR) selection. Cultures were diluted to ~0.5% parasitemia with fresh erythrocytes when parasitemia reached 5–10%. C. Episomally transfected P. falciparum parasites (NF54 +/-plasmid), which were initially selected under positive (WR) selection, and WT P. falciparum NF54 parasites were subjected to negative (5-FC 1μM final concentration). Cultures were diluted to ~0.5% with fresh erythrocytes when parasitemia reached 5–10%. (TIFF) [file pone.0168362.s001.tiff]

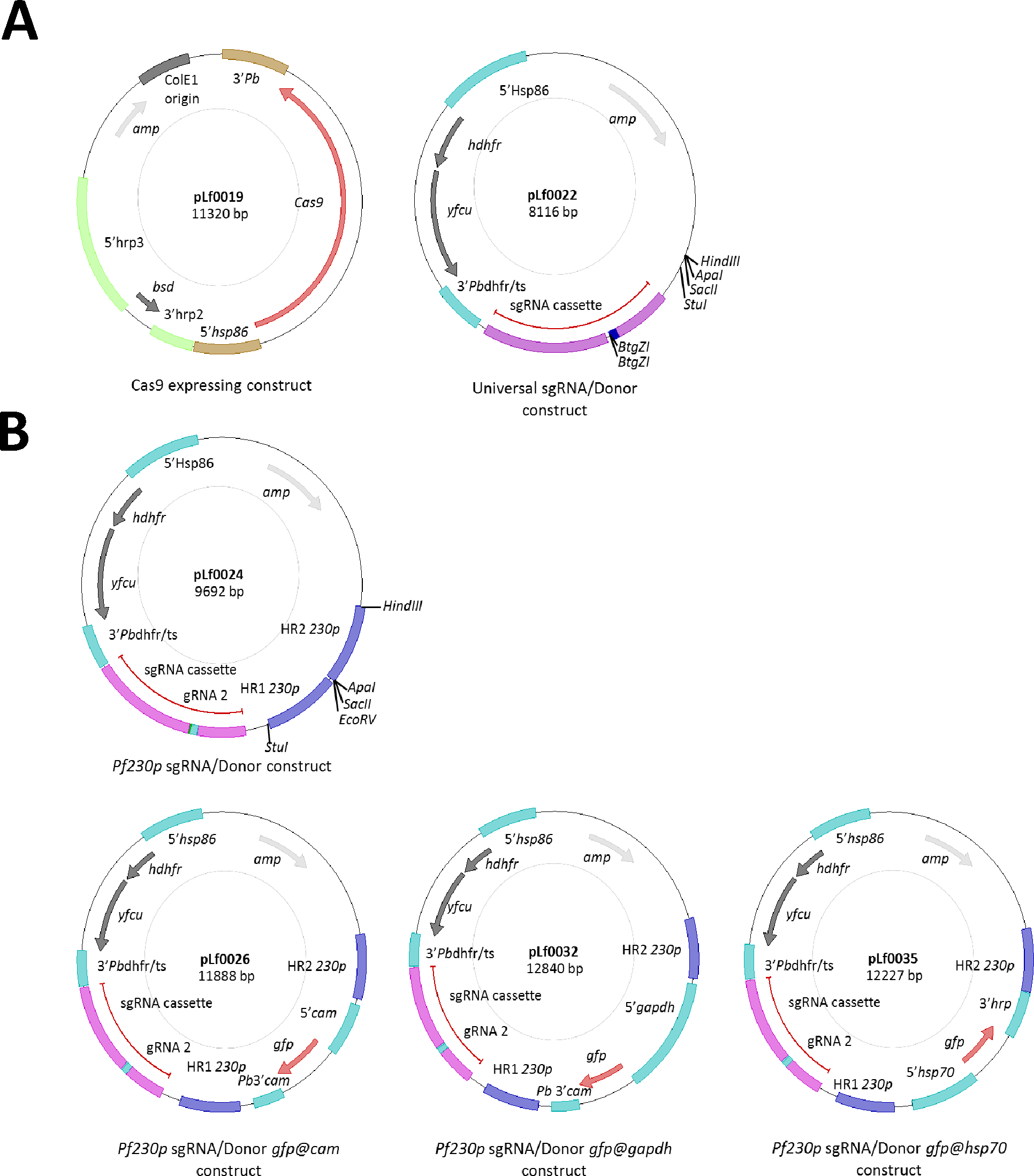

Supplement: S2 Fig — A. Basic constructs: pLf0019 for Cas9-expression construct with the bsd selectable marker; pLf0022 sgRNA/donor construct and pLf0024 for targeting the Pf230p locus. B. Constructs used for introduction of the GFP-expression cassettes into the P. falciparum genome: pLf0026 for gfp@cam, pLf0032 for gfp@gapdh and pLf0035 for gfp@hsp70 into Pf230p. (TIFF) [file pone.0168362.s002.tiff]

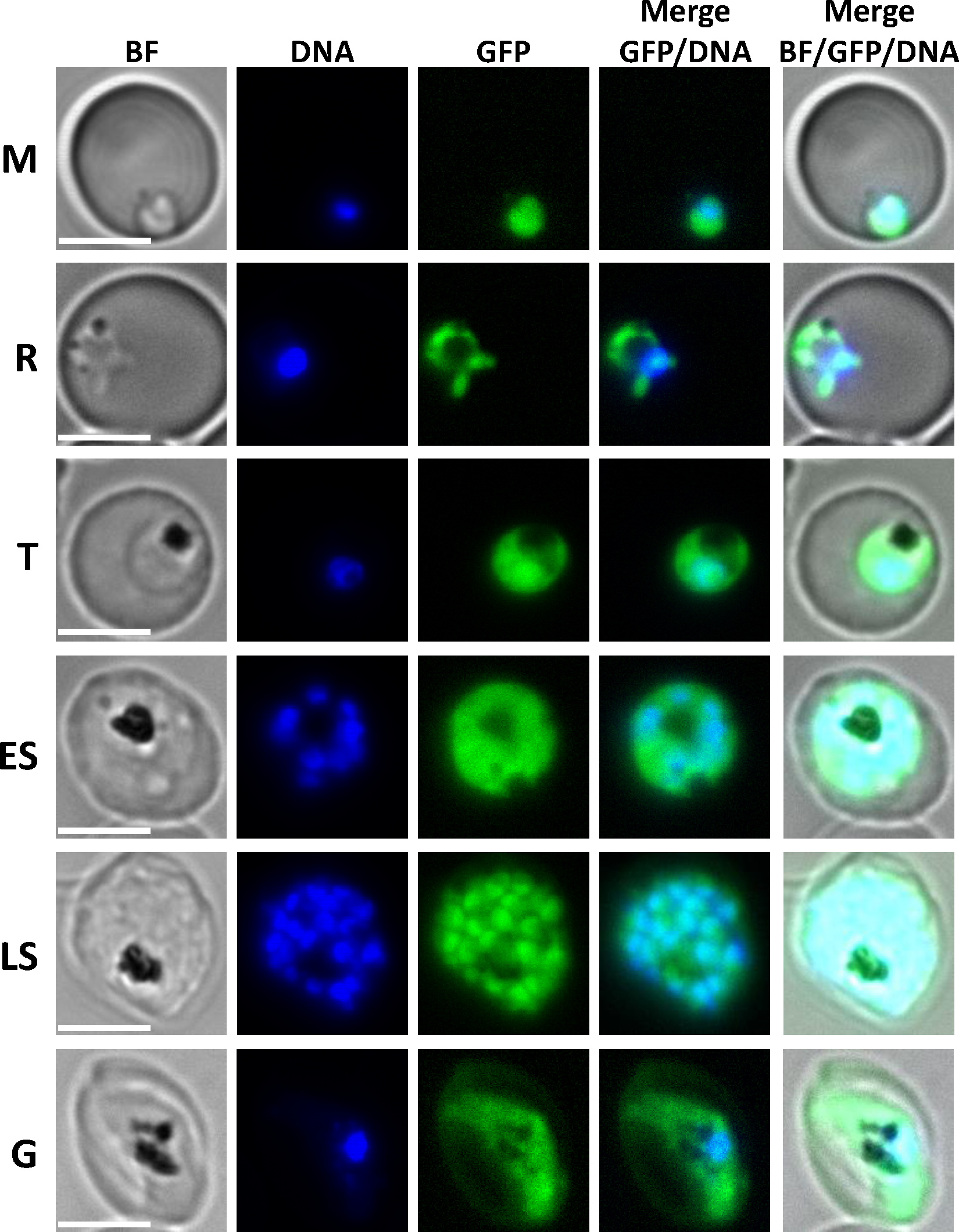

Supplement: S3 Fig — R: rings; T: trophozoites; ES: early schizonts; LS: late schizonts; G: gametocytes. Nuclei were stained with the DNA-specific dye Hoechst 33342. All pictures were recorded with standardized exposure/gain times to visualize differences in fluorescence intensity (GFP 0.7 s; Hoechst 0.136 s; bright field 0.62 s (1x gain)). (TIFF) [file pone.0168362.s003.tiff]

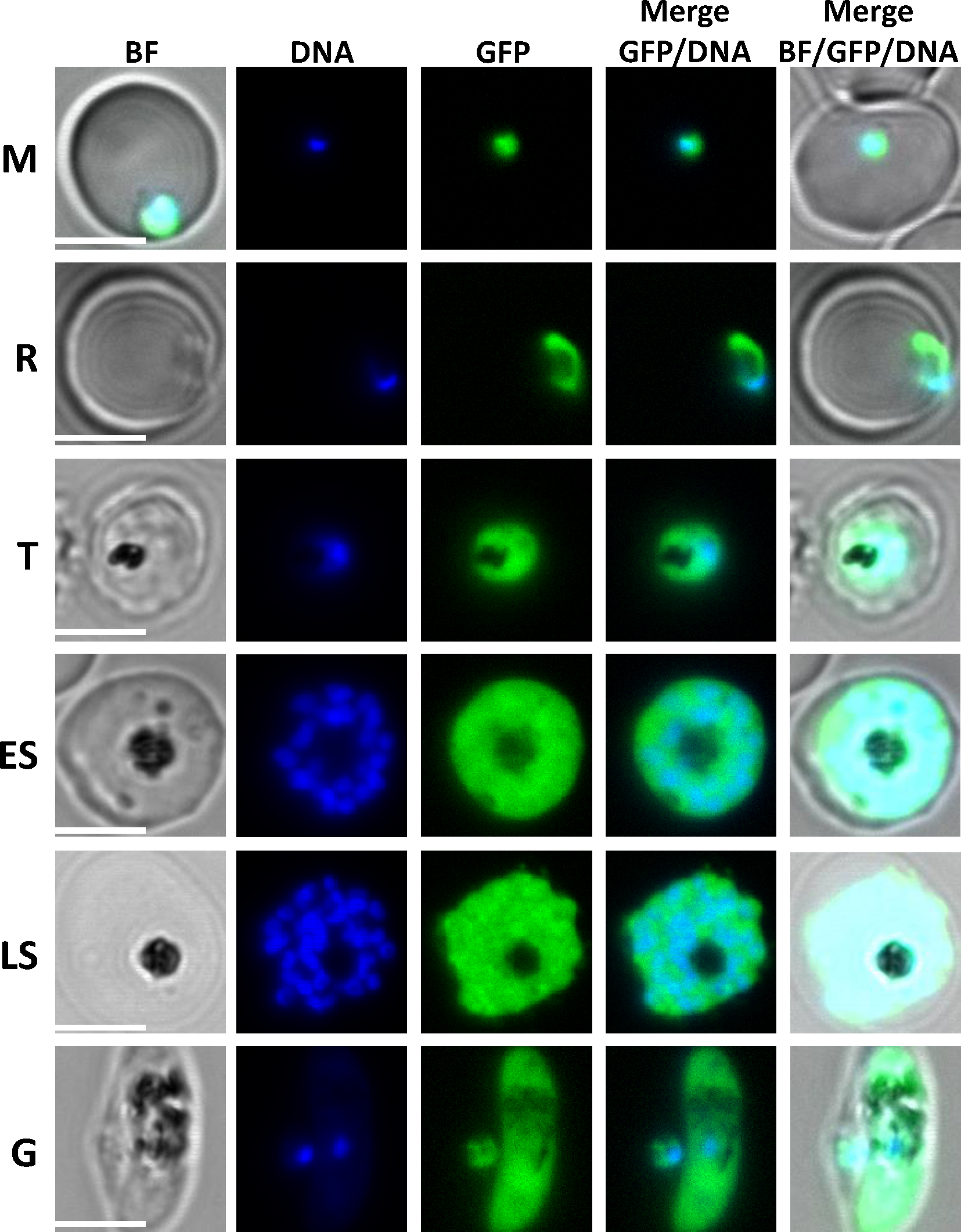

Supplement: S4 Fig — R: rings; T: trophozoites; ES: early schizonts; LS: late schizonts; G: gametocytes. Nuclei were stained with the DNA-specific dye Hoechst 33342. All pictures were recorded with standardized exposure/gain times to visualize differences in fluorescence intensity (GFP 0.7 s; Hoechst 0.136 s; bright field 0.62 s (1x gain)). (TIFF) [file pone.0168362.s004.tiff]

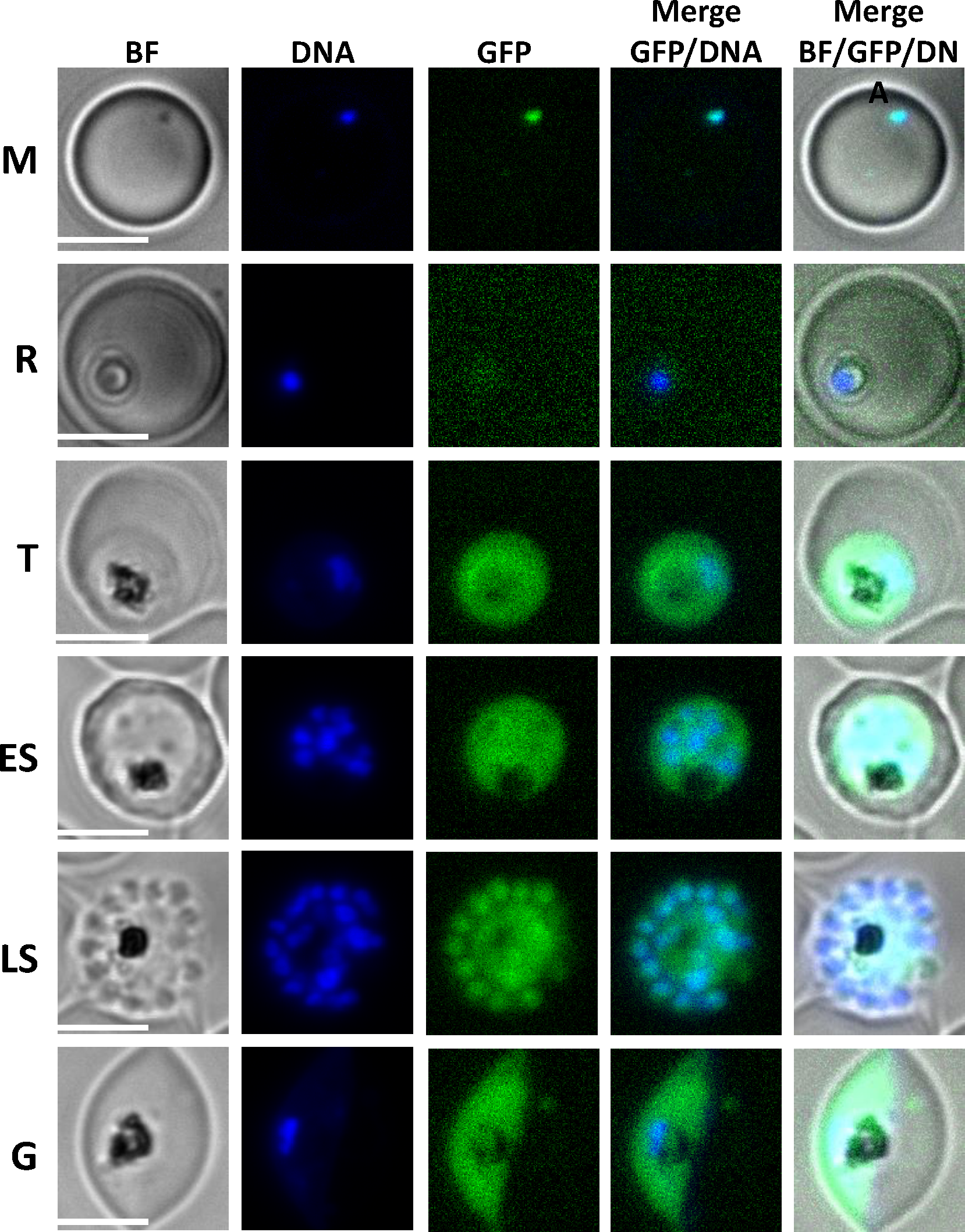

Supplement: S5 Fig — R: rings; T: trophozoites; ES: early schizonts; LS: late schizonts; G: gametocytes. Nuclei were stained with the DNA-specific dye Hoechst33342. All pictures were recorded with standardized exposure/gain times to visualize differences in fluorescence intensity (GFP 0.7 s; Hoechst 0.136 s; bright field 0.62 s (1x gain)). (TIFF) [file pone.0168362.s005.tiff]
