# Supplementary material for: Rapid Generation of Marker-Free P. falciparum Fluorescent Reporter Lines Using Modified CRISPR/Cas9 Constructs and Selection Protocol
Source: PLoS One. 2016 Dec 20;11(12):e0168362. doi: 10.1371/journal.pone.0168362 (PMC5172577; doi:10.1371/journal.pone.0168362)
Supplement: S1 Table — (DOCX) [file pone.0168362.s006.docx]

| **Primer ID** | **Leiden code** | **Gene ID** | **Sequence** | **Enzymes** | **Product (bp)** | **Description** |
| --- | --- | --- | --- | --- | --- | --- |
| ***Pf230p* deletion Homology Regions** | | | | | | |
| P1 | 7865 | PF3D7_0208900 | TAATTAGGCCTGCCGGCCATATTTATGTGACTTCTTAAAC | *Stu*I/*Nae*I | 848 | Forward HR 1 *pf230p* |
| P2 | 7867 | PF3D7_0208900 | TTCCTCCGCGGGATATCCAACCTTCTATTGGATTC | *Sac*II/*EcoR*V |  | Reverse HR 1 *pf230p* |
| P3 | 7869 | PF3D7_0208900 | TTATTGGGCCCGTCGACGTTGATAAGGATAGTGTTTCAG | *Apa*I/*Sal*I | 867 | Forward HR 2 *pf230p* |
| P4 | 7871 | PF3D7_0208900 | TCCTTAAGCTTTACGTAGGATTAATATTCCCATTAGG | *Hind*III/*SnaB*I |  | Reverse HR 2 *pf230p* |
| **sgRNA** | | | | | | |
| P7 | 7882 | PF3D7_0208900 | TAAGTATATAATATTGAATATTATTCTAATGATAAGTTTTAGAGCTAGAA |  | 50 | Forward sgRNA 2 |
| P8 | 7883 | PF3D7_0208900 | TTCTAGCTCTAAAACTTATCATTAGAATAATATTCAATATTATATACTTA |  |  | Reverse sgRNA 2 |
| **Promoters** | | | | | | |
| P11 | 7896 | PF3D7_1434200 | GTAATAGACGTCGGCCAAATAAGAAATATAAT | *Aat*II | 704 | Forward *calmodulin* promoter |
| P12 | 7897 | PF3D7_1434200 | ATCTGGATCCGATATATTTCTATTAGGTATTTATTATT | *BamH*I |  | Reverse *calmodulin* promoter |
| P13 | 7894 | PF3D7_1462800 | AACTATGACGTCGCTATGAAAAACATGGGTGTG | *AatI*I | 1657 | Forward *gapdh* promoter |
| P14 | 7895 | PF3D7_1462800 | AAATAGGATCCGAAAAGAATTAAAAAGCCGAAG | *BamH*I |  | Reverse *gapdh* promoter |
| P15 | 7733 | PF3D7_0818900 | AATAAGACGTCCGCATAAATATCTGGTGAAATACAAAC | *Aat*II | 968 | Forward *hsp70* promoter |
| P16 | 7734 | PF3D7_0818900 | AAATTCTCGAGGAACCTTTTGCACTAGCCAATTTTTC | *Xho*I |  | Reverse *hsp70* promoter |
| **3’ UTRs** | | | | | | |
| P17 | 7589 | PBANKA_1010600 | TTATTCAATTGACCGGTGGCCGCGACTCTAGAATTAT | *Mun*I/*Age*I | 448 | Forward 3’ *calmodulin* UTR *P. berghei* |
| P18 | 7706 | PBANKA_1010600 | AATTACCCGGGTCGCGACGGTACCGACCATATAAGAATTAAC | *Sma*I/*Nru*I |  | Reverse 3’ *calmodulin* UTR *P. berghei* |
| P19 | 7735 | PF3D7_0831800 | TTGTTTCTAGAGTTAACCTAGGGAAGTATATGAG | *Xba*I | 638 | Forward 3’ *histidin rich protein* UTR |
| P20 | 7736 | PF3D7_0831800 | TAAATGGGCCCCTTCGAATTCTGGATTTAATAAATATG | *Apa*I |  | Reverse 3’ *histidin rich protein* UTR |
| **Genotyping** | | | | | | |
| P21 | 7471 | PF3D7_0405300 | AGCCGCGGCATGGAGAAGGGTTCTATTTTATCG | *Sac*II | 5383 | Forward primer DNA control PCR *lisp2* |
| P22 | 7470 | PF3D7_0405300 | AACGCTAGCTTCCGGATCGCTGTCTTTAC | *Nhe*I |  | Reverse primer DNA control PCR *lisp2* |
| P23 | 7965 |  | GAACCCAAAGATTGTTTTTCAC |  |  | Forward Integration 1 GFP@*cam*  Forward integration 1 GFP@*gapdh*  Forward LRPCR GFP@*cam*  Forward LRPCR GFP@*hsp70* |
| P24 | 2547 |  | CGAGCTGGACGGCGACGTAAAC |  |  | Reverse Integration 1 GFP@*cam*  Reverse integration 1 GFP@*gapdh*  Forward *gfp* |
| P25 | 5515 |  | GCATGGACGAGCTGTACAAG |  |  | Forward Integration 1 GFP@*hsp70* |
| P26 | 7966 |  | GTAGATGAACTATTTAATAATACATGTGATTTAG |  |  | Reverse Integration 1 GFP@*hsp70* Reverse LRPCR GFP@*gapdh* |
| P27 | 2548 |  | CAGCAGGACCATGTGATCGCG |  |  | Reverse *gfp* |
| P28 | 7967 |  | GTATCTTTTAAATAATACGGTGTAACATC |  |  | Reverse LR-PCR GFP@*hsp70*  Reverse LR-PCR GFP@*cam* |
| P30 | 7964 |  | CTTATGAACGTACATCAGGAGAAG |  |  | Forward LR-PCR GFP@*gapdh* |
