# Supplementary material for: Rapid Generation of Marker-Free P. falciparum Fluorescent Reporter Lines Using Modified CRISPR/Cas9 Constructs and Selection Protocol
Source: PLoS One. 2016 Dec 20;11(12):e0168362. doi: 10.1371/journal.pone.0168362 (PMC5172577; doi:10.1371/journal.pone.0168362)
Supplement: S2 Table — Data obtained from PlasmoDB (www.plasmodb.org; published in Otto et al. (2010) Mol. Microbiol. 76(1):12–24). (DOCX) [file pone.0168362.s007.docx]

| **Time point** | ***eef1-a PF3D7_1357000*** | | ***cam PF3D7_1434200*** | | ***gapdh PF3D7_1462800*** | | ***hsp70 PF3D7_0818900*** | |
| --- | --- | --- | --- | --- | --- | --- | --- | --- |
|  | ***Non Unique**** | ***Unique*** | ***Non Unique**** | ***Unique*** | ***Non Unique**** | ***Unique*** | ***Non Unique**** | ***Unique*** |
| **0 h** | 1.766 | 215 | 0 | 335 | 0.000 | 1.413 | 1 | 3.668 |
| **8 h** | 2.143 | 283 | 0 | 226 | 0.000 | 2.519 | 1 | 3.211 |
| **16 h** | 2.548 | 297 | 0 | 178 | 0.092 | 2.663 | 3 | 4.100 |
| **24 h** | 5.909 | 642 | 0 | 256 | 0.000 | 6.900 | 4 | 6.378 |
| **32 h** | 4.329 | 581 | 0 | 561 | 0.000 | 12.811 | 1 | 4.975 |
| **40 h** | 1.435 | 132 | 0 | 366 | 0.177 | 3.201 | 1 | 1.188 |
| **48 h** | 1.371 | 130 | 0 | 330 | 0.158 | 3.086 | 1 | 1.165 |

*** Transcript levels of reads per kilobase of exon model per million mapped reads (RPKM).
Non-Unique sequences are shown to indicate the maximum expression potential of the analysed gene. *eef1-a* (*elongation factor 1α* ); *cam* (*calmodulin*); *gapdh* (*glyceraldehyde-3-phosphate dehydrogenase*); *hsp70* (*heat shock protein 70*)*.*

|  |
| --- |
|  |
|  |
|  |
